# Virus-driven remodeling of the immune microenvironment and response to immune checkpoint inhibitors: the infection– immunity–cancer crosstalk

**DOI:** 10.3389/fimmu.2026.1807408

**Published:** 2026-04-27

**Authors:** Guangying Wang, Feiyin Chen, Runda Zhao, Zhuoqi Liu, Xiaohong Yang, Daya Luo

**Affiliations:** 1 Queen Mary School, Jiangxi Medical College, Nanchang University, Nanchang, China; 2 The First Clinical Medical College of Nanchang University, Jiangxi Medical College, Nanchang University, Nanchang, China; 3 The MOE Basic Research and Innovation Center for the Targeted Therapeutics of Solid Tumors, Jiangxi Medical College, Nanchang University, Nanchang, China; 4 Jiangxi Provincial Key Laboratory of Tumor Biology, Nanchang University, Nanchang, China; 5 Department of Biochemistry and Molecular Biology, School of Basic Medical Sciences, Jiangxi Medical College, Nanchang University, Nanchang, China

**Keywords:** Epstein–Barr virus (EBV), hepatitis B virus (HBV), hepatitis C virus (HCV), human immunodeficiency virus (HIV), human papillomavirus (HPV), immune checkpoint inhibitors, viral reactivation, virus-associated tumors

## Abstract

Immune checkpoint inhibitors (ICIs) have revolutionized the therapeutic landscape of diverse solid tumors; however, concurrent viral infections significantly influence their efficacy and safety profiles. By driving persistent antigen exposure, inducing T cell exhaustion, and remodeling the immunosuppressive tumor microenvironment (TME), viruses extensively reconfigure tumor immune landscapes, leading to marked heterogeneity in responses to immunotherapy. Emerging evidence indicates that patients infected with hepatitis B virus (HBV), hepatitis C virus (HCV), human immunodeficiency virus (HIV), human papillomavirus (HPV), or Epstein–Barr virus (EBV) who receive ICIs therapy may not only regain antitumor immune function, but in some cases may also be associated with virological responses, immunological changes suggestive of improved viral control. However, the risk of viral reactivation remains a concern, particularly in the context of immune-related adverse events (irAEs) requiring immunosuppressive treatment. This review systematically summarizes the current clinical application of ICIs across different viral infection backgrounds and highlights recent advances in the underlying immunological mechanisms. Furthermore, we propose the potential value of virus-specific immune profiling in guiding individualized treatment strategies and emphasize the need to optimize the integration of ICIs and antiviral therapies from the perspective of systemic immune reprogramming.

## Introduction

In recent years, cancer immunotherapy has emerged as a transformative therapeutic strategy, achieving significant progress by harnessing the host immune system to eliminate tumor cells. Under physiological conditions, immune checkpoint pathways are essential for maintaining immune homeostasis and preventing autoimmunity (1). However, within the tumor microenvironment (TME), tumor cells exploit these pathways by upregulating the expression of immune checkpoint molecules to facilitate immune evasion (2). By intercepting programmed cell death protein 1 (PD-1)/programmed death-ligand 1 (PD-L1) or cytotoxic T-lymphocyte–associated antigen 4 (CTLA-4) signaling, immune checkpoint inhibitors (ICIs) reinvigorate T cell-mediated antitumor immunity. These agents have demonstrated durable and significant clinical efficacy across a wide range of malignancies, establishing themselves as a cornerstone of contemporary oncological treatment (3).

Notably, a growing body of evidence indicates that microbial factors, particularly viral infections, play a crucial role in shaping the TME and modulating host immune responses (4). For instance, infections such as hepatitis B virus (HBV), human papillomavirus (HPV), and Epstein–Barr virus (EBV) are not only intimately linked to the development and progression of specific tumors but also significantly influence the efficacy and safety of ICIs (5, 6). Clinical observations have revealed that certain cancer patients with concurrent viral infections may derive more substantial survival benefits from ICIs, while simultaneously facing potential risks such as viral reactivation (7). From a virological perspective, ICIs have also shown immunomodulatory and interventional potential in the context of chronic viral infections, such as HBV, hepatitis C virus (HCV), and human immunodeficiency virus (HIV) (8–10).

In view of the burgeoning body of clinical research on ICIs, particularly the accumulation of evidence in virus-associated malignancies, systematic reviews in this field remain relatively scarce. Existing literature largely focuses on the relationship between specific viral types and ICIs efficacy, lacking a systematic integration of the virus–immunity–cancer crosstalk from a pan-viral perspective. Furthermore, a comprehensive comparison of the underlying mechanisms, therapeutic variations, and safety profiles of ICIs across diverse viral contexts is yet to be established. Therefore, this review aims to bridge these gaps by systematically summarizing the clinical advancements and immunological mechanisms of ICIs in virus-associated malignancies from a holistic multi-viral perspective, while discussing their potential value in optimizing combination therapies and personalized immunotherapeutic strategies.

## Methods

This narrative review was conducted to summarize current clinical and mechanistic insights into the interactions between viral infections and ICIs therapy in cancer. A comprehensive literature search was performed using PubMed.

The search covered studies published from May 2006 to February 2026. The following key terms and their combinations were used: “immune checkpoint inhibitors”, “PD-1”, “PD-L1”, “CTLA-4”, “viral infection”, “virus-associated cancer”, “HBV”, “HCV”, “HPV”, “EBV”, “HIV”, “viral reactivation”, and “tumor microenvironment”.

Studies were screened based on their relevance to the topic of virus-associated malignancies and immunotherapy. Priority was given to high-quality clinical evidence, including prospective randomized clinical trials, phase I–III clinical trials, large retrospective studies, and real-world cohort analyses. Mechanistic studies and translational research exploring immune regulation and viral–tumor interactions were also included to provide biological context. Case reports and small case series were considered when they provided clinically relevant insights, particularly regarding viral reactivation or rare immune-related adverse events (irAEs).

Articles were excluded if they were not published in English, lacked sufficient methodological detail, or were not directly related to ICIs or virus-associated malignancies. During interpretation of the evidence, the hierarchy of available data was taken into account, with greater emphasis placed on prospective clinical trials, meta-analyses, and large cohort studies when available. Where multiple studies reported similar outcomes, representative studies with higher levels of evidence were prioritized.

### Human papillomavirus

HPV is a double-stranded DNA virus belonging to the family *Papillomaviridae*. HPV infection is highly prevalent worldwide and represents the fourth most common oncogenic factor currently identified, accounting for over 600,000 new malignancy cases annually. Epidemiological studies demonstrate that approximately 20%–30% of newly diagnosed head and neck squamous cell carcinoma (HNSCC) cases globally are associated with HPV infection; in oropharyngeal squamous cell carcinoma (OPSCC), this proportion can reach as high as 70%–80% (11, 12).

#### Therapeutic efficacy of ICIs in HPV-associated tumors

ICIs exhibit distinct clinical advantages in patients with HPV-positive HNSCC. Numerous studies have established that HPV-positive patients achieve higher objective response rates (ORR) and extended overall survival (OS) (13, 14). For instance, in the KEYNOTE-012 study an open-label, multicenter Phase 1b trial evaluating the anti-PD-1 antibody pembrolizumab in recurrent or metastatic HNSCC, the ORR was 24.8% in HPV-positive patients, compared with 13.3% in HPV-negative patients, with median progression-free survival (PFS) of 2.1 and 1.9 months, respectively (**Supplementary Table 1**) (15). A systematic analysis integrating 11 clinical trials, such as KEYNOTE-048 and KEYNOTE-055, further confirmed a median OS of 11.5 months in the HPV-positive population versus 6.3 months in the HPV-negative population (16). Furthermore, exploratory research by Theresa et al. reported that the survival curves of HPV-positive patients receiving immunotherapy display a characteristic “tail-plateau” effect, suggesting that this population may be more likely to achieve durable long-term survival benefits (17). The survival benefit of ICIs over conventional therapies, such as methotrexate, docetaxel, or cetuximab, becomes more evident in randomized controlled trials. Specifically, the CHECKMATE-141 trial demonstrated that nivolumab improved the median OS from 4.4 to 9.1 months in HPV-positive HNSCC patients (HR 0.55, 95% CI 0.35–0.86) (**Supplementary Table 1**) (18–20).

This difference in clinical outcomes may be partly explained by the unique immunogenicity of HPV-associated tumors. The persistent expression of the oncogenic proteins E6 and E7 in HPV-associated tumors provides stable virus-derived tumor antigens, which can enhance antigen presentation and immune recognition. This process contributes to increased infiltration of tumor-infiltrating lymphocytes (TILs), including exhausted CD8^+^ T cell subsets whose function can be partially restored by ICIs (21–24). Single-cell sequencing studies have further revealed significant differences in the gene expression profiles of CD4^+^ T cells and B cells between HPV-positive and HPV-negative tumors (25), suggesting marked heterogeneity in the composition of the tumor immune microenvironment between these two tumor types. Moreover, HPV-positive tumors exhibit relatively intact antigen presentation machinery and higher activation of the cGAS-STING pathway, which further bolsters innate and adaptive immune responses (26). Concurrently, the widespread upregulation of inhibitory molecules such as PD-1, PD-L1, and CTLA-4 in HPV-positive OPSCC—believed to be driven by E6/E7-mediated cytokine release and the activation of NF-κB/JAK-STAT signaling—provides potential therapeutic targets for ICIs-induced immune reinvigoration (23, 27–32). Furthermore, in a retrospective analysis of a cohort comprising 328 patients treated with anti-PD-1 or anti-PD-L1 monoclonal antibodies, Vanhersecke et al. observed that 84 HNSCC patients with mature tertiary lymphoid structures (TLS) exhibited superior responses to ICIs therapy (ORR of 62%). Conversely, patients with myeloid-enriched profiles had significantly shorter PFS (HR = 2.31, 95% CI 1.28–4.18), suggesting that TLS maturity may serve as a critical biomarker for predicting ICIs efficacy, independent of CD8^+^ T cell density. Notably, HPV-positive tumors are typically more predisposed to forming mature TLS (33, 34). This indicates that, in addition to the T cell inflamed phenotype, TLS formation is another pivotal antitumor immune mechanism. As reported by Ruffin et al., transcriptional signatures of germinal center phenotype tumor-infiltrating B cells (TIL-Bs) and the presence of germinal center -containing TLS are associated with significantly improved clinical outcomes in patients with HPV-positive HNSCC (34). These TLS can enhance antitumor immune responses by sustaining the clonal expansion of tumor antigen-specific B cells and promoting T–B cell synergy, thereby potentiating the sensitivity of HPV-positive tumors to ICIs.

#### Safety of ICIs in HPV-associated tumors

The safety profile and irAEs of ICIs in patients with HPV-positive HNSCC remain a primary focus of clinical concern. Given that HPV infection can trigger DNA damage, chronic inflammation, and genomic instability, some studies suggest that HPV-positive patients may experience more pronounced immune-related toxicities when receiving ICIs or combination regimens involving chemoradiotherapy (35–38). However, current clinical evidence has not demonstrated a clear association between HPV status and the overall incidence of irAEs. For example, in the CheckMate-141 trial, HPV status was not significantly associated with the overall incidence of irAEs (**Supplementary Table 1**) (39). Nevertheless, disparities may exist in specific types of irAEs, such as thyroid dysfunction, which may be partly attributed to the more frequent history of neck radiation in HPV-positive patients (39, 40). In a safety stratification analysis by Zeng et al., which pooled data from over 3,000 patients, the incidence of Grade 3–4 adverse events was significantly lower in the ICIs group than in the chemotherapy group; however, no statistically significant differences were observed between HPV-positive and HPV-negative subgroups regarding discontinuation rates or mortality due to adverse events (41). This may be explained by the fact that HPV-induced immune infiltration is primarily confined to the TME; although the local tumor exhibits a “hot” phenotype, it does not necessarily imply a state of systemic immune hyperactivation in the peripheral circulation (41). Consequently, HPV status is primarily regarded as a predictive biomarker for therapeutic efficacy rather than a reliable predictor of irAEs (41). Notably, a study (n = 30) investigating toripalimab combined with concurrent chemoradiotherapy for locally advanced HPV-associated cervical cancer reported that 80% (24/30) of patients experienced Grade 3 or higher treatment-related adverse events (TRAEs), including leukopenia, lymphopenia, and anemia (**Supplementary Table 1**) (42–44). This suggests that when ICIs are integrated with conventional therapies, HPV-positive patients may require vigilant monitoring for cumulative toxicity risks (42–44). Given the small sample size, these findings should be interpreted with caution and require further validation in larger clinical studies. Nevertheless, the study suggests that potential additive toxicities should be carefully considered when ICIs are combined with conventional therapies.

#### The role of HPV in resistance to ICIs

Although some patients derive significant clinical benefit from ICIs, approximately 80% of patients with HPV-associated recurrent or metastatic tumors either fail to respond to PD-1 inhibitors or eventually develop secondary resistance (23, 45, 46). The primary mechanisms underlying this resistance are intimately linked to the immunosuppressive remodeling of the TME driven by HPV infection.

HPV-infected tumor cells secrete various immunomodulatory factors (e.g., IL-6, IL-10, and TGF-β), which promote the recruitment of myeloid-derived suppressor cells (MDSCs) and regulatory T cells (Tregs), thereby suppressing effector T cell function and facilitating immune evasion (32, 45–48). Furthermore, CD8^+^ T cell heterogeneity is prominent in HPV-positive tumors. Cheng et al. identified a specific subset of proliferative exhausted CD8^+^ T cells associated with a favorable prognosis; however, the transcriptional signatures of these cells may be suppressed following PD-1/PD-L1 blockade, thereby affecting the therapeutic response (49, 50). Genomic alterations in the HPV virus also influence immune recognition. While HPV integration and E6/E7 gene methylation can lead to resistance through antigen loss, aneuploidy and genomic rearrangements may drive neoantigen production, thereby increasing the tumor neoantigen load and further intensifying immune heterogeneity (27, 32, 35, 51). Additionally, HPV exosome-mediated TME remodeling, T cell functional exhaustion, downregulation or loss of MHC-I molecules on tumor cells, and aberrant activation of the JAK/STAT pathway may contribute to reduced efficacy of ICIs (31, 36, 49, 52–54).

It is currently believed that the development of secondary resistance to ICIs in patients with HPV-positive HNSCC is associated with MHC-I deficiency. Under the selective pressure of ICIs therapy, tumor cells may undergo genetic mutations or epigenetic modifications, leading to the acquired downregulation or loss of MHC-I expression (55). Alternatively, mutations in the β2-microglobulin (B2M) gene can result in the functional impairment of MHC-I complexes (56, 57). Furthermore, JAK1/2 mutations may lead to defective IFN-γ signaling, rendering tumor cells resistant to IFN-γ-induced PD-L1 expression and MHC-I upregulation, thereby driving secondary resistance to ICIs (58).

Recent studies have revealed a synergistic upregulation of multiple checkpoint molecules in resistant HPV-positive tumors. The co-expression of TIM-3, LAG-3, and VISTA in CD8^+^ cells reached 71.5% in the resistant group, which was significantly higher than the 28.3% observed in the sensitive group 57, suggesting that co-inhibition by multiple checkpoints may be a pivotal mechanism underlying ICIs resistance in HPV^+^ patients. Other molecules contributing to immunosuppression include CTLA-4 and TIGIT; under such multifaceted inhibitory conditions, monotherapy targeting the PD-1/PD-L1 axis may be insufficient to overcome the immunosuppressive milieu (46, 57, 59).

Notably, T cell exhaustion is not entirely irreversible. T cells in the early stages of reversible exhaustion, primarily governed by the PD-1/PD-L1 pathway, can still regain effector functions through PD-1/PD-L1 blockade. However, T cells in states of profound exhaustion or terminal differentiation undergo extensive remodeling of their epigenetic and transcriptomic landscapes, rendering them insensitive or even entirely non-responsive to PD-1/PD-L1 inhibition (60–63).

**Figure 1:**
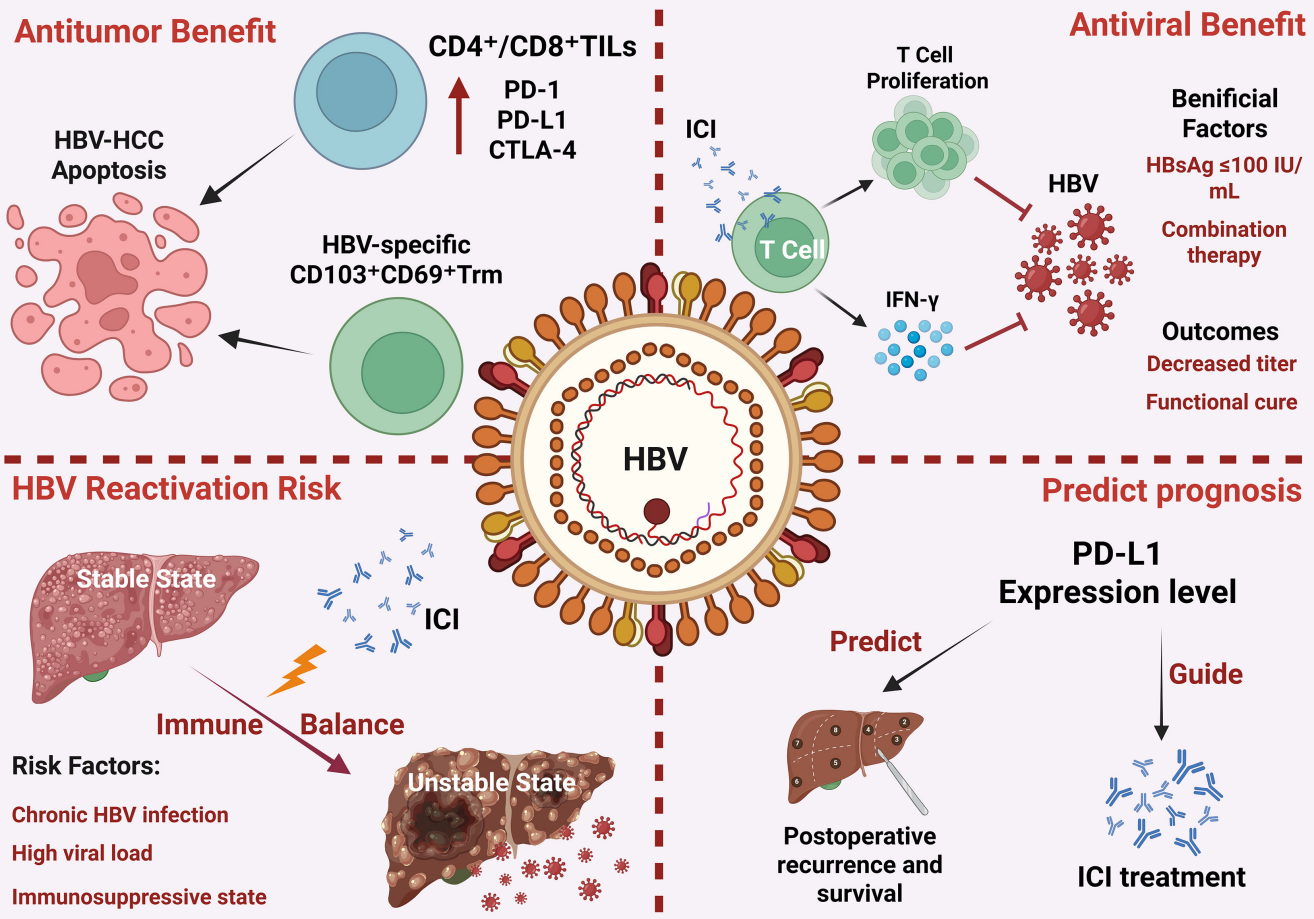
Overall effects of ICIs in patients with HPV infection.

**Figure 1** illustrates the overall effects and underlying mechanisms of ICIs in the treatment of patients with HPV infection. Regarding therapeutic efficacy, HPV E6/7 proteins enhance ICIs response by increasing TIL infiltration, activating inflammatory pathways, and promoting the formation of TLS, concomitantly with the upregulation of PD-1, PD-L1, and CTLA-4 expression. In terms of irAEs, HPV-induced genomic instability and chronic inflammation may synergize with the effects of ICIs, potentially leading to hematologic toxicities such as leukopenia, lymphopenia, and anemia. The development of resistance is primarily driven by an immunosuppressive TME (mediated by MDSCs, Tregs, and their secreted factors including TGF-β, IL-6, IL-10, IL-13, IL-18, and CCL28), alterations in tumor antigens, exosome-mediated interference, and the upregulation of alternative immune checkpoints such as TIM-3 and LAG-3.

### Epstein–Barr virus

EBV, also designated as human herpesvirus 4 (HHV-4), is a member of the gammaherpesvirus family. EBV is intimately linked to the pathogenesis of various malignancies, including Burkitt lymphoma (BL), nasopharyngeal carcinoma (NPC), and gastric cancer (GC) (64). By expressing viral oncoproteins such as LMP1 and EBNA1, EBV facilitates tumorigenesis and progression through the activation of signaling cascades—including NF-κB, JAK/STAT, and PI3K/Akt (65)—disruption of cell cycle regulation (66), alteration of host immune recognition (67), and remodeling of the TME (68). EBV-associated tumors are frequently characterized by the upregulation of immune checkpoint molecules such as PD-L1, suggesting that ICIs may serve as a pivotal therapeutic modality for these patients (6, 69).

#### Therapeutic efficacy of ICIs in EBV-associated tumors

In EBV-associated lymphomas—including BL with a latency III phenotype, diffuse large B-cell lymphoma (DLBCL) (particularly EBNA2-driven subtypes), and natural killer/T cell lymphoma (NKTL)—tumor cells frequently exhibit marked upregulation of the PD-1/PD-L1 pathway, rendering them ideal targets for ICIs (69, 70). A study involving seven patients with relapsed/refractory NKTL demonstrated a 100% ORR (n=7) with pembrolizumab treatment, including five complete responses (CR) characterized by a comprehensive decline in radiological findings, pathological evidence, and circulating EBV DNA levels (**Supplementary Table 1**) (**Table 1**) (70). Although the sample size of this study was limited, the findings suggest that ICIs may have potential therapeutic value in EBV-associated lymphomas. Combination therapeutic strategies have further enhanced efficacy; for instance, a Phase II trial (n=28) utilizing a regimen of avelumab priming plus R-CHOP (the standard-of-care regimen comprising rituximab, cyclophosphamide, doxorubicin, vincristine, and prednisone) followed by avelumab consolidation for DLBCL reported a two-year failure-free survival (FFS) of 82% and an OS of 89%. Notably, a higher ORR was observed in the EBV-positive DLBCL subgroup (**Supplementary Table 1**) (**Table 1**) (71, 72). However, given the small sample size and the limited number of EBV-positive patients (only three cases with clearly defined EBV-positive molecular features), these findings require further validation in larger studies. Furthermore, research on ICIs in EBV-associated NPC has progressed rapidly in recent years, demonstrating substantial clinical potential. Both anti-PD-1/PD-L1 and anti-CTLA-4 monotherapies have shown favorable safety, tolerability, and antitumor activity, while combination strategies involving diverse ICIs can further augment therapeutic outcomes (**Supplementary Table 1**) (**Table 1**) (73, 74). A study of 40 pretreated patients with NPC (39 of whom had received prior chemotherapy) revealed that combined PD-1 and CTLA-4 blockade yielded a best overall response rate (BOR) of 38% (95% CI: 23.5–54.4) and a disease control rate (DCR) of 80%, with Grade ≥3 adverse events occurring in only 25%—the treatment exhibited an acceptable safety profile (**Supplementary Table 1**) (**Table 1**) (75). This study also indicated that patients with baseline plasma EBV-DNA <7,800 IU/mL achieved superior therapeutic outcomes and longer PFS, suggesting that viral load is intimately correlated with the degree of T cell exhaustion and immunosuppression. Furthermore, the presence of PD-1^+^/CTLA-4^+^ CD8^+^ T cell subsets was associated with enhanced clinical benefit, serving as a potential predictive biomarker (75). The TME of EBV-associated NPC is highly distinctive. The largest integrated single-cell and spatial transcriptomic study of NPC to date revealed that these tumors can form high-quality TLS that produce EBV-specific plasma cells. These cells not only directly induce apoptosis in virus-infected tumor cells but also potentiate anti-PD-1-induced CD8^+^ T cell responses by increasing the exposure of EBV antigens (76). The study further characterized NPC as a malignancy driven by a multicomponent immune network involving B cells, T cells, TLS, and fibroblasts, in which the functional quality of TLS, rather than their quantity alone, may be a key determinant of response to PD-1 blockade (63).

**Table 1:** Overview of the clinical efficacy and safety of ICIs in EBV-associated tumors.

Cancer type	Viral latency/biomarker	ICIs treatment (target)	Efficacy (ORR/survival/HR)	Safety (key irAEs)	Ref
BL	Latency III-expressing BLs	Anti-PD-L1 (69)		Not reported	(69)
DLBCL	EBV^+^ DLBCL	Anti-PD-L1 (69)		Not reported	(69)
		Acitumab+Rituximab →R-CHOP→Acitumab (72)	FFS: 82% OS: 89% (72)	Not reported	(72)
NKTL	Relapsed/refractory NKTL	Pembrolizumab (PD-1) (70)	ORR: 7/7 CR: 5/7 (70)	No Grade ≥3 AEs	(70)
PTLD	EBV^+^ (Latency III) PD-L1 Often High	Nivolumab / Pembrolizumab (Salvage Therapy)	ORR: ~30-40%	Acute graft rejection and loss of function (72)	(72)
NPC	Refractory NPC	Cadonilimab (PD-1/CTLA-4), Nivolumab + Ipilimumab	(PD-L1 expression ≥44.4%) (74) 12-month OS rate: 79.7% (74) ORR: 38% (75) DOR: 12.6 months (75)	2/23: Grade≥3 irAEs; Common irAEs: hypothyroidism (30.4%), rash (21.7%), and pruritus (21.7%) 25%: Grade≥3 AEs (lower than in other malignancies) Treatment discontinuation due to AEs: n=5 (75)	(75)
GC	EBV^+^ GC	Pembrolizumab (77)	ORR: 100% (77) PFS: 10.3 months; OS: 28.1 months (78)	Not reported	(77) (78)
		PD-1/PD-L1/CTLA-4 (108)	OR = 8.47 (108)	Not reported	(108)

BL, Burkitt Lymphoma; DLBCL, Diffuse large B-cell lymphomas; PTLD, Post-transplant lymphoproliferative disorder; NPC, Nasopharyngeal Carcinoma; GC, Gastric Cancer; PD-1, Programmed Death-1; PD-L1, Programmed Death Ligand-1; OS, overall survival; PFS, progression-free survival; FFS, failure-free survival; NKTL, Natural killer/T cell lymphoma; DOR, duration of response; ORR, objective response rate; CR, complete response; R-CHOP, rituximab, cyclophosphamide, doxorubicin, vincristine, and prednisone; AEs, adverse events; irAEs, immune-related adverse events.

This table summarizes the key clinical findings of ICIs (encompassing anti-PD-1, PD-L1, and CTLA-4 antibodies) across various types of EBV-associated malignancies. The data are categorized by tumor type and subtype, detailing therapeutic regimens, ORR, survival outcomes (PFS/OS), and safety signals (irAEs). Notably, meta-analyses have demonstrated that EBV-positive GC exhibits an immunotherapeutic response significantly superior to that of EBV-negative tumors (OR = 8.47). Additionally, small-cohort studies in NKTL have shown high response rates, whereas ICIs treatment in PTLD is associated with a substantial risk of acute graft rejection.

Although GC is characterized by high incidence and mortality rates, the overall response rate to PD-1 inhibitors remains low and exhibits significant variability (approximately 8%–26%), highlighting the critical clinical value of identifying reliable predictive biomarkers. In a cohort study of 61 patients, all EBV-positive cases (n = 8) achieved a PR. Moreover, PD-L1 positivity was found to be strongly associated with EBV positivity and MSI-H status, suggesting that EBV-positive GC may represent another subgroup, similar to MSI-H tumors, that could potentially benefit from immunotherapy (**Supplementary Table 1**) (**Table 1**) (77). In a first-line treatment cohort of 293 patients with advanced GC receiving nivolumab plus chemotherapy, EBV-positive cases accounted for only 12 patients; however, this subgroup showed improved clinical outcomes compared with EBV-negative patients. Specifically, the PFS of EBV-positive patients was nearly doubled (10.3 vs. 5.7 months), and the disparity in OS was even more pronounced (28.1 vs. 15.0 months), suggesting that EBV infection may contribute to more durable antitumor immune responses (**Supplementary Table 1**) (**Table 1**) (78). In locally advanced gastric cancer (LAGC) or adenocarcinoma of the gastroesophageal junction (AEGJ), perioperative ICIs-based combination therapy can significantly improve cure rates and long-term survival in patients with a PD-L1 combined positive score (CPS) ≥5 or high EBV expression. Furthermore, integrating ICIs with multimodal therapeutic regimens may improve pathological response rates and long-term clinical outcomes, representing a promising strategy for converting immunologically “cold” tumors into more responsive phenotypes and enhancing the benefits of immunotherapy (79).

However, some evidence suggests that EBV-positive status does not invariably guarantee a favorable response to ICIs. For instance, certain patients with EBV-positive DLBCL exhibit suboptimal responses, which may be attributed to the high expression of PD-1 on Tregs, leading to a “paradoxical suppression” of the immune response (80). Consequently, pretreatment immunophenotypic and virological stratification to identify potential responders represents a pivotal direction for future precision medicine. However, current studies investigating the response of EBV-positive DLBCL to ICIs are largely based on small cohorts, and the level of evidence remains limited. Therefore, these findings require further validation in larger prospective studies.

#### Safety of ICIs in EBV-associated tumors

Although numerous studies have validated the safety of ICIs in EBV-associated tumors, reports of associated irAEs remain noteworthy, particularly in specific populations with fragile immune homeostasis where vigilant monitoring is essential.

In the context of EBV-associated post-transplant lymphoproliferative disorder (PTLD), animal studies have demonstrated that PD-1/PD-L1 blockade can result in total graft loss in transplant models (81). Furthermore, certain models suggest that ICIs may paradoxically promote the growth of EBV-positive PTLD (81). Therefore, achieving a balance between antitumor efficacy and the risk of acute graft rejection in these patients remains an important clinical challenge (71, 82).

With the widespread clinical application of ICIs in solid tumors, the incidence of associated gastrointestinal toxicities (ICIGI) has progressively increased, with the role of viral infection or reactivation gaining increasing prominence. EBV reactivation is commonly defined as the detection of EBV DNA in plasma by PCR (83). Multiple case reports have observed that ICIs-related colitis is frequently associated with infections such as HBV or EBV (**Supplementary Table 1**) (83, 84), suggesting that the disruption of mucosal immunity may create a permissive environment for viral reactivation. Histological analysis indicates that approximately 13% of ICIGI patients harbor EBV infection, occasionally accompanied by cytomegalovirus (CMV) or adenovirus co-infection (84). Notably, in two patients with solid tumors who developed severe and refractory immune-mediated colitis, colon biopsies revealed significantly elevated EBV loads (138,261 IU/mL and 2,425,556 IU/mL, respectively); their symptoms improved markedly following antiviral treatment with ganciclovir (**Supplementary Table 1**) (85). However, current evidence is mainly derived from case reports or small cohort studies, and the precise role of EBV reactivation in ICIs-related gastrointestinal toxicity remains to be further elucidated. Therefore, future studies should strengthen virological monitoring and further clarify the clinical significance of viral reactivation in ICIs-associated immune toxicity.

### Hepatitis B virus

Hepatitis B virus (HBV) is a primary etiological factor for hepatocellular carcinoma (HCC), accounting for approximately 50% of HCC cases worldwide (86). As a DNA virus, HBV drives hepatocarcinogenesis through diverse mechanisms, including the enhancement of host genomic instability, the activation of oncogenic signaling pathways, and the remodeling of the hepatic immune microenvironment (87).

#### Therapeutic efficacy of ICIs in HBV-associated tumors

Provided that standardized antiviral therapy (SAT) is maintained as fundamental management, evidence from translational research and clinical cohorts increasingly suggests that HBV infection does not impair the antitumor efficacy of ICIs in HCC. Conversely, some studies indicate that patients with HBV-associated HCC may derive survival benefits from ICIs that are comparable to, or even superior to, those of their non-viral counterparts (**Supplementary Table 1**) (**Table 2**) (88, 89). Numerous retrospective and real-world studies have consistently demonstrated that ICIs therapy reduces the risk of mortality and disease progression while prolonging PFS and OS in the HBV-associated HCC population. Du et al. analyzed eight studies involving 5,646 patients to evaluate the differential efficacy of ICIs among HCC patients with HBV, HCV, or non-viral etiologies. Their findings revealed that ICIs were associated with a 29% reduction in the risk of death and a 47% reduction in the risk of disease progression in HBV-positive patients (**Supplementary Table 1**) (**Table 2**) (88). Furthermore, a 2024 meta-analysis found that HBV-positive HCC patients exhibited a more favorable prognosis compared to non-infected individuals, characterized by a statistically significant prolongation of both PFS (6.7 vs. 5.8 months) and OS (17.15 vs. 15.2 months) (**Supplementary Table 1**) (**Table 2**) (89). Furthermore, research has indicated that ICIs combination therapies yield superior efficacy compared to monotherapy. Shao et al. evaluated the STRIDE regimen—comprising durvalumab (anti-PD-L1) plus tremelimumab (anti-CTLA-4)—against durvalumab monotherapy, observing that the combination therapy resulted in superior ORR (10% VS 25%) (**Supplementary Table 1**) (**Table 2**) (90), suggesting that dual immune checkpoint blockade may compensate for the limitations of single-agent therapy. The immunological underpinnings of this differential response are being progressively uncovered. Cheng et al. identified HBV-specific CD103^+^CD69^+^ tissue-resident memory T cells (Trm) in HBV-positive HCC, which exhibit low PD-1 expression and maintain plasticity, thereby avoiding the fixed state of terminal exhaustion governed by the PD-1 pathway. PD-1/PD-L1 blockade (durvalumab) significantly bolsters the effector functions of these HBV-Trm cells and correlates with favorable clinical outcomes, while tremelimumab (anti-CTLA-4) acts during the early T cell priming stage to expand the intrahepatic HBV-specific T cell repertoire. However, disparities in the priming mechanisms between HBV-specific and antitumor T cells may limit the adequacy of standalone PD-1 blockade (91). Additionally, other research has demonstrated upregulated PD-1 expression in both CD4^+^ and CD8^+^ TILs from HBV-positive HCC patients, where targeted PD-1 inhibition can reinvigorate their antitumor activity (91).

**Table 2:** Comparison of efficacy, safety, and antiviral effects of ICIs therapy among patients with HBV-associated, HCV-associated, and non-viral malignancies.

Outcomes		HBV	HCV	Non-viral infection
Oncological efficacy	OS	HR 0.71 (88); HR 0.81 (ICIs monotherapy) (109); HR 0.65 (ICIs + VEGF/VEGFRi) (109) 17.15 months (IQR: 14.3–20 months) (89); 6.7 months (90) 21.7 vs. 12.8 months (lenvatinib plus sintilimab vs. lenvatinib) (105)	HR 0.80 (88); HR 0.79 (ICIs monotherapy) (109); HR 0.72 (ICIs + VEGF/VEGFRi) (109); 16.8 months (89)	HR 0.87 (88); HR 0.83 (ICIs monotherapy); HR 0.98 (ICIs + VEGF/VEGFRi) (109)
	PFS	HR 0.53 (88); 6.7 months (IQR: 5–8.4 months) (86); 2.3 months (110); 11.3 vs. 6.6 months (lenvatinib plus sintilimab vs. lenvatinib) (105)	HR 0.65 (74); HR 0.70 (109); 8.35 months (89)	HR 0.81 (not statistically significant) (88); PFS: 5.8 months (IQR: 5.48–6.13 months) (86)
Adverse events	Total AEs	Hepatitis, hypertension, fatigue, diarrhea, HFSR, abdominal pain, nausea, and anorexia (105)	Pneumonitis, colitis, hepatitis, and hypothyroidism (111)	Gastrointestinal, dermatological, musculoskeletal, and endocrine (112)
	≥Grade 3	5/90 (Lenvatinib) (105); 6/60 (Lenvatinib plus sintilimab); 1/15 (ropeg + nivolumab); 4/30 (90); 3/15 (113); hepatitis 23/511 (114); 29/162 (115)	8/52 (hepatitis: 5/52 (9)); 2/40 (111)	Anti-PD-1 mAb: 10%–15%; anti-CTLA-4 mAb: 20%–30%; anti-CTLA-4 plus anti-PD-1 combination therapy: 55% (116)
Antiviral potential	Reduction	Cancer patients: HBsAg ≥ 10 IU/mL HBsAg reduction: 16.3% (at 24 months, baseline <100 IU/mL) vs. 0% (at 24 months, baseline ≥100 IU/mL) (93) HCC patients: HBsAg reduction ≥100 IU/mL in 2/15 (92); ≥1 log HBsAg reduction: 38.4% (at 24 months, baseline <100 IU/mL) vs. 0% (at 24 months, baseline ≥100 IU/mL) (93); 3/6 (117)	Cancer patients: HCV RNA decreased >10 IU/mL: 4/52 (9)	-
	Clearance	Cancer patients: 5/32 VS 3/71 (<100UI/ml VS ≥100UI/ml) (93) HCC patients: HBsAg clearance: 3/15 (92); 4/18 VS 0/17 (<100UI/ml VS ≥100UI/ml) (93)	Cancer patients: 2/52 (9)	-
Viral reactivation	ALT elevation	3/89 (≥grade 3) (110)	17/98 (≥grade 3) (110)	-
	Increased viral load	44/307 (95); 1/60 (117); 26/633 (118); ICIs with prophylaxis: HBVr 4/387 (119); ICIs without prophylaxis: HBVr 2/10 (119); 1/24 (120); 5/511 (HCC: 2/409 vs. others: [3/102]) (114); 16/218 (anti-PD-1 plus angiogenesis inhibitors vs. anti-PD-1 monotherapy: 14 vs. 2) (121); 7/162 (115)	2/52 (9); 2/387	-
Other findings		Elevated HBV titers are indicative of poor prognosis	HCVr secondary to the administration of immunosuppressive agents for the management of ICIs-related irAEs	–
References		(86, 88–90, 92, 95, 105, 109, 110, 113–115, 117–121)	(9, 88, 90, 109–111)	(86, 88, 109, 112, 116)

OS, overall survival; PFS, progression-free survival; AEs, adverse events; irAEs, immune-related adverse events; HR, hazard ratio; IQR, interquartile range; HBsAg, hepatitis B surface antigen; VEGF, vascular endothelial growth factor; VEGFRi, vascular endothelial growth factor receptor inhibitor; ropeg, ropeginterferon alfa-2b; HFSR, hand-foot skin reaction; HBVr, HBV reactivation.

This table provides a comprehensive comparison of clinical outcomes between cancer patients with chronic HBV or HCV infection and those with non-viral etiologies following ICIs therapy. The data indicate that patients with HBV- or HCV-associated malignancies often derive comparable or even superior survival improvements (HR 0.53–0.81) relative to non-viral cases, without a significant increase in non-hepatic serious adverse events. However, the latter portion of the table elucidates the complex virus–immunity crosstalk: following the alleviation of immunosuppression by ICIs, both antiviral effects (e.g., >1 log reduction in HBsAg titers and HCV RNA decline) and risks of viral reactivation (e.g., ALT elevation and viral load rebound) are observed. Notably, the risk of HBVr is significantly elevated in the context of combined anti-angiogenic therapy or the absence of antiviral prophylaxis. These findings underscore the necessity for longitudinal monitoring of viral load and liver function to balance the benefits of antitumor immune activation against the risks of viral reactivation in patients with virus-associated malignancies (See **Supplementary Table S1** for **Supplementary Information**).

#### Potential impact of ICIs on HBV replication and antiviral immune responses

Beyond their antitumor effects, ICIs may also play a role in remodeling host antiviral immunity. Emerging evidence suggests that these agents may help restore HBV-specific immune responses (**Supplementary Table 1**) (**Table 2**) (92), with possible effects on HBV replication control. Mon et al. demonstrated that among cancer patients receiving ICIs, those with low baseline HBsAg titers (<100 IU/mL) were more likely to achieve HBsAg seroclearance, suggesting that ICIs may accelerate viral clearance by revitalizing host antiviral immune activity (93). In the HBV-positive HCC population, a single-arm Phase II study involving 30 patients with active chronic hepatitis B (CHB) who were not on antiviral therapy (mean baseline HBV DNA ≈ 7.7 × 1,000,000 IU/mL) showed that HBV DNA levels dropped below 2,000 IU/mL in all patients before the third dose of nivolumab (**Supplementary Table 1**) (**Table 2**) (90). These findings suggest that, under specific immunological conditions, ICIs therapy does not necessarily induce viral reactivation and may instead be associated with reduced viral replication. However, due to the limited sample size and the absence of a control group, these observations should be interpreted cautiously. Basic and *in vitro* immunological research provides mechanistic support for these findings: HBV-specific CD8^+^ T cells in the peripheral blood of chronic HBV patients typically exhibit high PD-1 expression, and their exhaustion state correlates positively with viral load. Blockade of the PD-1 pathway enhances IFN-γ secretion and T cell proliferation, thereby restoring antiviral activity (94). In addition, in patients with CHB with controlled viral replication, PD-L1 blockade appears to be safe and may further contribute to a decline in HBsAg levels, particularly in those with baseline HBsAg ≤100 IU/mL (8, 95). Notably, the immunological processes underlying HBsAg decline or clearance may not be triggered by ICIs in isolation. In a Phase I trial evaluating postoperative sequential therapy with ropeg interferon alfa-2b (IFN-α) and anti-PD-1 for HBV-associated HCC, 5 out of 15 patients experienced alanine aminotransferase (ALT) flares accompanied by HBsAg reduction or clearance, with these events occurring predominantly after the transition from IFN-α to anti-PD-1. This phenomenon suggests that IFN-α may initially disrupt viral homeostasis and activate innate immunity, whereas subsequent PD-1 blockade alleviates the suppression of HBV-specific CD8^+^ T cells, thereby promoting viral clearance (**Supplementary Table 1**) (92). In clinical practice, an important challenge is to distinguish the underlying causes of ALT/aspartate aminotransferase (AST) elevations during ICIs therapy, including ICIs-related immune-mediated hepatitis, HBV reactivation (HBVr), or a “hepatitis flare” associated with immune reconstitution. The latter may signify a robust and favorable antiviral immune response. Consequently, for patients with low baseline viral loads and a declining HBsAg trend, cautious continuation of ICIs therapy under intensive monitoring (e.g., frequent assessment of HBV DNA and liver function) may represent a rational strategy, rather than a categorical discontinuation of treatment. In the clinical management of liver disease, a pivotal concern is whether transient elevations in ALT/AST during ICIs therapy signify immune-mediated clearance rather than hepatotoxicity. Prematurely discontinuing treatment without integrating both immunological and virological contexts may interrupt the ICIs-induced immune process targeting HBV. It must be emphasized that although case reports and small-cohort studies suggest that ICIs may reduce HBV DNA or even HBsAg levels, prospective studies have yet to confirm the achievement of a stable functional cure. Such occurrences may parallel immune reconstitution inflammatory syndrome (IRIS); therefore, vigilance regarding the concomitant risk of liver injury remains imperative.

#### Safety of ICIs in HBV-associated tumors

Although numerous studies have validated the overall safety of ICIs in cancer patients with HBV infection (**Supplementary Table 1**) (7, 96, 97), a potential risk of inducing HBVr persists. According to the latest guidelines from the American Association for the Study of Liver Diseases (AASLD), the diagnostic criteria for HBVr include: (I) an increase in HBV DNA≥100 IU/mL from baseline; (II) an increase in HBV DNA≥1,000 IU/mL in patients with undetectable baseline levels; or (III) an HBV DNA level≥10,000 IU/mL when baseline data are unavailable (98). Although the incidence of HBVr is relatively low, it can lead to hepatic injury and, in severe cases, may progress to liver failure, typically accompanied by elevated ALT levels (99). This risk is intimately associated with chronic HBV infection, high viral load, and immunosuppressive states. Chronic viral hepatitis itself serves as a primary risk factor for liver-related adverse events during ICIs therapy (100); notably, the risk of HBVr is significantly elevated in patients with baseline HBV DNA levels ≥100,000 IU/mL, HBeAg positivity, and white blood cell (WBC) counts <4×10^9^/L (101). A meta-analysis involving 2,561 patients demonstrated that the risk of HBVr in chronic HBV carriers is significantly higher than in those with resolved infection (OR = 8.69, 95% CI 2.16–34.99). Moreover, patients who did not receive antiviral prophylaxis faced a tenfold higher probability of reactivation compared to those receiving prophylaxis (OR = 0.12, 95% CI 0.02–0.67) (**Supplementary Table 1**) (102). All HBsAg-positive patients, regardless of HBV DNA detectability, should initiate prophylactic antiviral therapy (e.g., entecavir or tenofovir) prior to ICIs treatment, alongside regular monitoring of HBV DNA and liver function. However, the underlying mechanisms of ICIs-induced HBVr remain largely elusive. Evidence suggests that chronic HBV infection leads to exhausted immune responses characterized by the upregulation of the PD-1/PD-L1 axis (103). While PD-1 physiologically prevents excessive immune-mediated hepatic damage, its blockade may disrupt the immune equilibrium established during chronic infection, triggering hepatocyte destruction and viremia, which ultimately culminates in HBVr (104). Fundamentally, HBVr represents a dysregulation of viral control following immune reconstitution, serving as a “double-edged sword” during the restoration of the immune system. Therefore, systematic screening of HBV status before ICIs therapy and serial monitoring of ALT and HBV DNA levels are imperative for the early detection of HBVr or immune-mediated liver injury. This review further underscores the necessity of antiviral prophylaxis for all HBsAg-positive patients undergoing immunotherapy to mitigate HBV-associated adverse events.

In HBV-positive cancer patients receiving effective antiviral prophylaxis, current evidence has not shown a significant increase the risk of Grade ≥3 severe irAEs. Low-grade irAEs are common but generally well-tolerated, typically requiring neither treatment discontinuation nor corticosteroid intervention. Common TRAEs include rash, hepatitis, hypertension, fatigue, diarrhea, hand-foot skin reaction (HFSR), abdominal pain, nausea, and anorexia (**Supplementary Table 1**) (**Table 2**) (105). In this population, distinguishing between ICIs-induced immune-mediated hepatitis and HBV-related liver injury is paramount. While both conditions can present with elevated ALT levels, the latter is typically characterized by a synchronous increase in HBV DNA, whereas immune-mediated hepatitis occurs without a concomitant rise in viral replication. HBV-related liver injury can be effectively managed with antiviral agents and usually does not necessitate the interruption of ICIs; conversely, ICIs-induced immune-mediated hepatitis is unpreventable, carries a higher clinical risk, and often requires treatment cessation and the initiation of immunosuppressive therapy (106). Under chronic HBV infection, the TME undergoes remodeling, allowing immune checkpoint molecules to exert multifaceted roles in immune regulation beyond mere immune evasion. Studies have demonstrated that high PD-L1 expression is significantly associated with poor prognosis in HBV-positive HCC patients following curative resection. Lin et al. further indicated that PD-L1 serves as an independent predictor of postoperative recurrence and survival in HBV-positive HCC. Notably, ICIs therapy targeting the PD-1/PD-L1 pathway holds promise for reversing this unfavorable prognostic trend, offering new avenues for precision immunotherapy in HBV-related liver cancer (107).

#### Clinical management recommendations

All HBsAg-positive patients should initiate preemptive antiviral therapy prior to the commencement of ICIs and continue for at least 6–12 months following ICIs discontinuation. During the treatment course, longitudinal monitoring of HBV DNA levels and liver function indicators is recommended every 4–12 weeks. In the event of ALT elevation, prompt etiological differentiation is imperative to guide subsequent clinical decision-making.

**Figure 2:**
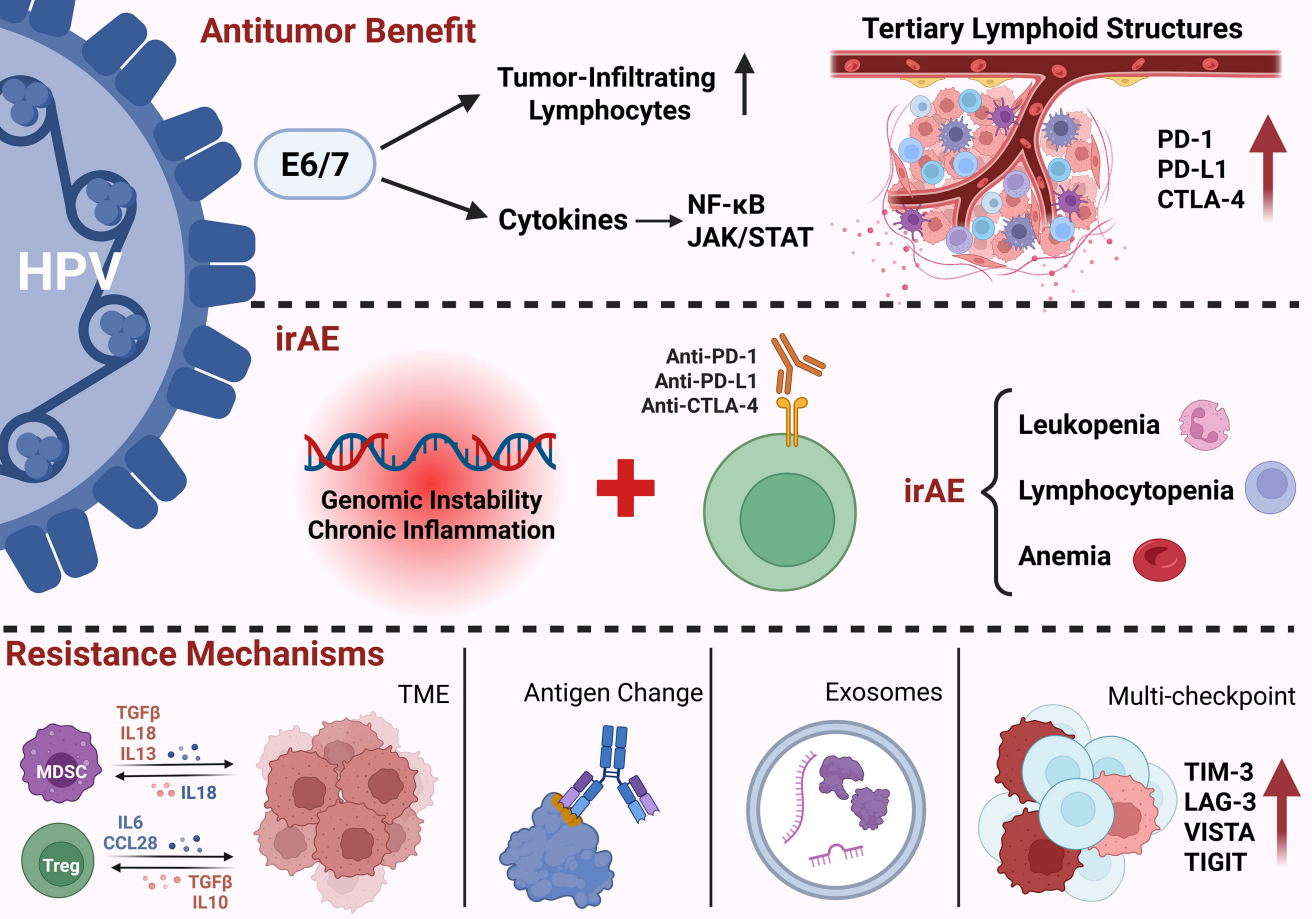
The quadruple effects of ICIs in patients with HBV infection.

**Figure 2** systematically illustrates the quadruple effects of ICIs in patients with HBV infection. Regarding antitumor efficacy, ICIs therapy significantly increases the infiltration and activation of CD4^+^/CD8^+^ TILs and HBV-specific CD103^+^CD69^+^ Trm cells by alleviating immunosuppression, thereby inducing apoptosis in HBV-associated HCC cells. ICIs also exhibit potential for HBV suppression; this therapy inhibits viral replication by promoting T cell proliferation and IFN-γ secretion. Particularly in patients with low baseline HBsAg levels (≤100 IU/mL) or those receiving combination strategies, ICIs may be associated with reductions in viral markers; however, their contribution to a functional cure remains uncertain. However, the treatment is also associated with a risk of HBVr. In high-risk populations characterized by chronic HBV infection, high viral loads, or immunosuppressed states, ICIs may disrupt the existing immune homeostasis, leading to hepatic instability and triggering viral reactivation. Finally, key immunological indicators possess significant prognostic value. PD-L1 expression levels in hepatic tissue not only serve as biomarkers for predicting postoperative recurrence and survival but also provide guidance for the development of personalized ICIs treatment regimens.

### Hepatitis C virus

HCV is a single-stranded, positive-sense RNA virus belonging to the family *Flaviviridae* and the genus *Hepacivirus* (110). Since its initial identification in 1989, HCV infection has emerged as a major global etiological factor for chronic hepatitis and HCC (122). The World Health Organization (WHO) estimates that approximately 71.1 million individuals worldwide are chronic HCV carriers, with an annual incidence of approximately 1.75 million new infections (123).

#### Therapeutic efficacy of ICIs in HCV-associated tumors

Several lines of evidence suggest that HCV-positive cancer patients may exhibit enhanced sensitivity to ICIs therapy. In an etiology-stratified systematic review and network meta-analysis, ICIs monotherapy demonstrated significant survival improvements compared to sorafenib in HCV-associated HCC (HR for OS 0.79, 95% CI 0.64–0.98) (**Table 2**). Furthermore, the network meta-analysis from the same study indicated that immunotherapeutic combination strategies targeting the vascular endothelial growth factor (VEGF) pathway—particularly atezolizumab plus bevacizumab—achieved higher efficacy rankings in the HCV population. Pairwise analyses further confirmed that the combination of ICIs and VEGF/vascular endothelial growth factor receptor (VEGFR) inhibitors prolonged PFS (HR 0.70, 95% CI 0.53–0.92), indicating a potential benefit in disease control (**Supplementary Table 1**) (**Table 2**) (109). Real-world evidence further supports this trend. A retrospective study involving 19 patients with non–small cell lung cancer (NSCLC) and concurrent prior/chronic HBV or chronic HCV infection showed favorable overall PFS in the HCV-positive cohort following ICIs treatment. Specifically, patients achieving a partial response (PR) experienced durable efficacy for ≥10–12 months, while those with stable disease (SD) typically maintained PFS for 1–5 months (113). These findings suggest that HCV-positive patients can safely receive ICIs, and their therapeutic sensitivity may even surpass that of patients with non-viral etiologies.

#### Potential impact of ICIs on HCV replication and antiviral immune responses

Similar to observations in HBV, declines in HCV RNA levels have also been observed during ICIs therapy in some HCV-infected patients who had not received direct-acting antiviral (DAA) treatment. However, it must be emphasized that for HCV infection, achieving a cure through DAA therapy is prioritized and should serve as the foundation before initiating ICIs. Ideally, DAA treatment should be completed to achieve a sustained virologic response (SVR) prior to immunotherapy to minimize the risks of viral interference and liver injury. Current research defines HCV suppression as a reduction in HCV RNA of more than 10 IU/mL from baseline following ICIs therapy in the absence of concurrent DAA treatment. Studies have reported that virologic suppression occurs in approximately 7.7%–12% of antiviral-naive HCV patients after receiving ICIs (124). Yibirin et al. systematically evaluated the virologic and biochemical dynamics of 52 chronic HCV-positive patients with solid tumors following ICIs treatment, revealing that approximately 11.5% of patients experienced a significant decline in HCV RNA, with two cases achieving undetectable HCV RNA. Importantly, ALT levels returned to the normal range in all patients who achieved viral suppression or clearance, suggesting that ICIs can partially reverse HCV-specific T cell exhaustion, thereby restoring antiviral immune responses and exerting direct or indirect anti-HCV effects (9).

#### Safety of ICIs in HCV-associated tumors

Current evidence suggests that HCV infection is not a contraindication for ICIs therapy. In patients receiving standard antiviral treatment or those with effectively controlled viral replication, ICIs are generally well-tolerated, with no significantly increased risk of viral reactivation or hepatic decompensation being observed. Based on pooled evidence from 11 studies encompassing 387 patients with solid tumors and concurrent HCV, the incidence of HCV reactivation (HCVr) is remarkably low (0.5%) in patients treated with DAAs or those with well-controlled viral loads. Furthermore, all reported HCVr events occurred in patients receiving intensive immunosuppressive therapy for severe irAEs rather than being directly induced by ICIs (124). HCVr is defined as an increase in HCV RNA of ≥1 log 10 IU/mL from baseline following ICIs treatment. Compared with HBVr, the clinical course of HCVr is typically more indolent and less likely to lead to severe hepatic injury or decompensation (9). In the study by Yibirin et al., the only two reported cases of reactivation occurred in patients receiving potent immunosuppressants for severe irAEs, with peak ALT levels reaching 613 and 492 U/L, respectively. This indicates that the trigger for HCVr is not the ICIs themselves, but rather the loss of antiviral T cell control secondary to immunosuppression. Although immunotherapy-related HCVr events are rare, current literature suggests that they occur almost exclusively in patients receiving systemic immunosuppression (moderate-to-high-dose corticosteroids ± other immunosuppressive agents) for severe irAEs (**Table 2**). Collectively, ICIs exhibit a favorable safety profile in HCV-infected patients; hepatic impairment is primarily associated with immune-mediated hepatitis or viral rebound following immunosuppression rather than direct drug-induced HCV flares (9, 111, 124). Across various malignancies, the incidence of ICIs-related adverse events remains comparable to that reported for HCV-negative patients in clinical trials, regardless of concurrent antiviral therapy. This suggests that HCV infection does not significantly augment the risk of adverse reactions during ICIs treatment (111).

Consequently, for patients presenting with moderate-to-severe irAEs who require intensive immunosuppression, enhanced virological monitoring is warranted. Antiviral strategies should be initiated if clinically indicated to mitigate the risk of HCVr and ensure therapeutic safety (125, 126).

### Human immunodeficiency virus

HIV is a retroviral RNA virus that predominantly targets host CD4^+^ T lymphocytes, leading to progressive immune dysfunction and a significantly elevated risk of various malignancies (127).

#### Therapeutic efficacy of ICIs in HIV-infected patients

Numerous studies have demonstrated that ICIs are both safe and effective in HIV-infected patients with malignancies (**Supplementary Table 1**) (128–130). Following ICIs treatment, response rates and survival outcomes in HIV-infected individuals are comparable to those observed in HIV-negative patients. In a single-center controlled study, 18 treatment-naive HIV-positive patients with NSCLC and 40 HIV-negative controls received tislelizumab plus platinum-based chemotherapy followed by maintenance therapy. The results indicated no statistically significant differences between the two groups regarding ORR (77.8% vs. 77.5%), 6-month PFS (83.3% vs. 82.5%), and 6-month OS (88.9% vs. 97.5%), suggesting that HIV infection does not impair the antitumor efficacy of ICIs (**Supplementary Table 1**) (131). The CATCH-IT multicenter retrospective study further established the broad applicability of ICIs in HIV-infected populations. Among 390 patients, 94% had an HIV viral load <400 copies/mL and 70% had CD4^+^ counts ≥200 cells/µL (**Supplementary Table 1**) (130); in matched analyses, there were no statistically significant differences in long-term PFS and OS between HIV-positive and HIV-negative patients with advanced NSCLC.

#### Potential effects of ICIs on HIV immune responses and viral reservoirs

Beyond their antitumor effects, ICIs have also been explored for their potential to modulate HIV-related immune responses. Several studies suggest that blockade of the PD-1/PD-L1 pathway may partially restore HIV-specific T cell function and influence the dynamics of the latent HIV reservoir, thereby providing new insights into potential immunotherapeutic strategies for HIVT cell (132). Neil J. Shah et al. observed reductions in HIV viral loads in certain patients (e.g., from 111,000 to 7,960 copies/mL and from 56,572 to 82 copies/mL). Chronic HIV infection leads to T cell exhaustion and the sustained upregulation of immune checkpoint molecules, such as PD-1, CTLA-4, LAG-3, and TIGIT (133–135), which has been associated with the maintenance of the latent HIV reservoir. Existing research indicates that ICIs can partially restore HIV-specific T cell function and may induce the expression of latent viruses, thereby enhancing antiviral immune responses. This immune restoration effect may be more pronounced when multiple immune checkpoints are targeted simultaneously (10, 136). Consequently, ICIs may represent not only a safe antitumor strategy for HIV-infected patients but also a promising immunological tool for a functional HIV cure. It should be noted that current evidence regarding the effects of ICIs on the HIV reservoir or viral replication is primarily derived from case reports or small cohort studies, and the findings should therefore be interpreted with caution.

#### Safety of ICIs in HIV-infected patients

In a pooled analysis of 73 HIV-infected patients, over 85% of whom received anti-PD-1 therapy, the incidence of Grade ≥3 irAEs was only 8.6%. Among patients with available follow-up data, 93% maintained an undetectable viral load, and CD4^+^ T cell counts exhibited a mild increase in the majority of cases, demonstrating favorable immunological stability. The ORR across various malignancies was 30% for NSCLC, 27% for melanoma, and 63% for Kaposi sarcoma (128). The CATCH-IT multicenter retrospective study further noted that among the most common malignancies (NSCLC, HCC, and HNSCC), the overall incidence of irAEs was approximately 20%, with Grade ≥3 irAEs at 7.7%, which is comparable to HIV-negative control populations (130). Notably, patients with a CD4^+^:CD8^+^ ratio >0.4 had a higher incidence of irAEs, suggesting that a more activated immune state may heighten the risk of immune-related toxicities. Furthermore, another study identified risk factors for severe irAEs, including low absolute CD4^+^ counts, CMV seropositivity, a history of major surgery, and a longer duration since HIV diagnosis (130).

In contrast to HBV and HCV, the primary risk for HIV-infected patients receiving ICIs is not viral reactivation but rather the heightened susceptibility to opportunistic infections (OIs) superimposed on their underlying immunodeficiency. Although ICIs are generally safe, their administration remains a significant risk factor for the development of irAEs. The management of these irAEs typically necessitates the use of immunosuppressive agents, such as corticosteroids or other immunosuppressants. However, these agents can significantly increase the risk of OIs, a concern that is particularly critical for immunocompromised HIV-infected individuals. Therefore, during the management of irAEs in HIV-infected cancer patients, enhanced monitoring, prevention, and treatment of OIs are essential (137). Evidence suggests that the degree of infection risk primarily depends on the patient’s immune status. Close monitoring of viral load and CD4^+^ T cell counts, alongside the continuous or prompt initiation of combination antiretroviral therapy (cART), is imperative to maintain immune homeostasis, mitigate infection risk, and improve overall prognosis. Future research should focus on the stratification of immune status in HIV-infected individuals and the potential synergy between antiretroviral therapy and ICIs (138).

## Discussion

This review endeavors to re-evaluate the biological characteristics and therapeutic opportunities of virus-associated malignancies in the ICIs era within the holistic framework of the “infection–immunity–cancer crosstalk.” Historically, in clinical practice, these patients have been categorized as “special populations” or “high-risk groups” regarding ICIs therapy, with clinical attention primarily centered on safety, viral reactivation, and the management of irAEs. However, our understanding of therapeutic disparities and the optimization of treatment strategies remains limited. Consequently, this article seeks to facilitate a paradigm shift in clinical thinking—moving from “whether ICIs should be used” to “how to use ICIs optimally.” Specifically, we emphasize the importance of integrating antiviral therapy, immune monitoring, and ICIs strategies across diverse viral contexts to maximize antitumor benefits while rigorously mitigating risks.

### The general efficacy of ICIs in virus-associated malignancies

Across the spectrum of HPV, EBV, HBV, HCV, and HIV-associated malignancies, recurring immunological signatures can be discerned despite significant disparities in their infection modes, tissue tropisms, and oncogenic mechanisms (**Table 3**). Persistent exposure to viral antigens typically establishes a chronic inflammatory milieu, further driving T cell exhaustion and the remodeling of immunosuppressive networks. Within this context, ICIs may unleash substantial antitumor immune potential. A primary consideration is the viral antigen burden and its spatial distribution. HPV and EBV infections are predominantly localized within the tumor, creating an environment with a concentrated high antigen density. These tumors are frequently characterized by robust intratumoral T cell infiltration and the formation of TLS, rendering their overall immunophenotype more akin to “hot tumors” (20, 64). In contrast, HBV, HCV, and HIV primarily manifest as systemic chronic infections, where hepatic or lymphoid tissues serve as major viral reservoirs. Consequently, the tumor itself often represents the culmination of a prolonged background of systemic immune dysfunction (139, 140). Furthermore, the degree of T cell exhaustion and its potential reversibility play central roles. In HPV- or EBV-associated malignancies, progenitor exhausted T cells with stem-like characteristics can still be detected; these cells retain a degree of proliferative capacity and functional reinvigoration potential, serving as a critical foundation for ICIs efficacy (63, 89). Conversely, during chronic HBV or HCV infections, virus-specific T cells are more prone to entering a state of profound exhaustion, characterized by the co-expression of multiple immune checkpoints. This population serves as the primary target for ICIs while also explaining the coexistence of therapeutic efficacy and immune-related toxicities (103, 127). HIV infection presents an even more intricate scenario: PD-1-high CD4^+^ memory T cells simultaneously constitute the viral reservoir and participate in the immune response, necessitating a delicate balance between the potential clearance of the reservoir and the induction of hyper-immune activation upon ICIs intervention. Additionally, the background of tissue-specific tolerance is a decisive factor. Different viral targets possess distinct immune microenvironments—such as the liver, lymph nodes, and upper respiratory mucosa—that are inherently predisposed toward immune tolerance under homeostatic conditions. This specialized microenvironment largely dictates the state of virus-driven inflammation. In the liver, for example, Kupffer cells, liver sinusoidal endothelial cells, hepatic stellate cells, and Tregs collectively establish a highly tolerant niche that facilitates HBV/HCV persistence. The application of ICIs disrupts this balance, which can either bolster antitumor and antiviral effects or trigger immune-mediated hepatitis and even hepatic decompensation (141, 142). In summary, distinct virus-associated malignancies exhibit significant disparities in TME landscapes, ICIs sensitivity, and combination therapeutic strategies. Tumors characterized by high antigen burden, robust immune infiltration, and functional plasticity—such as HPV- and EBV-associated malignancies—tend to demonstrate greater sensitivity to ICIs. Conversely, in malignancies dominated by persistent antigen exposure, immune tolerance, and states of profound exhaustion (e.g., in the context of HBV, HCV, or HIV), more sophisticated combination or stratified therapeutic approaches are required to effectively optimize immunotherapeutic outcomes.

**Table 3:** Comparative immunological mechanisms and immune checkpoint inhibitor responses across major virus-associated malignancies.

Virus	Major tumor types	Key viral oncogenic mechanisms	Immune microenvironment characteristics	Immune checkpoint features	ICIs response pattern	Safety / viral reactivation risk
HPV	HNSCC, cervical cancer	E6/E7 oncoproteins drive tumorigenesis and antigen presentation	High TIL infiltration, abundant CD8^+^ T cells, TLS formation	PD-1/PD-L1 and CTLA-4 upregulation	Generally favorable responses to ICIs	irAEs similar to HPV-negative patients
EBV	NPC, GC, lymphomas	LMP1, EBNA proteins activate NF-κB, JAK/STAT pathways	Immune-rich TME with B cells, plasma cells, and TLS	High PD-L1 expression frequently observed	Often sensitive to PD-1 blockade	Limited evidence; viral reactivation rarely reported
HBV	HCC	Chronic inflammation and viral integration induce genomic instability	Exhausted HBV-specific T cells and altered hepatic immune tolerance	PD-1 upregulation on CD8^+^ T cells	Comparable or slightly improved outcomes with ICIs	Risk of HBVr; antiviral prophylaxis recommended
HCV	HCC	Chronic inflammation and immune dysregulation	T cell exhaustion and altered cytokine milieu	PD-1 pathway activation in chronic infection	ICIs show clinical benefit in HCV-associated HCC	HCVr uncommon
HIV	Kaposi sarcoma, NSCLC, other malignancies	Chronic immune activation and CD4^+^ T cell depletion	T cell exhaustion and persistent viral reservoir	Multiple checkpoint molecules upregulated	ICIs appear effective in selected patients	OIs risk during immunosuppression

HPV, human papillomavirus; EBV, Epstein–Barr virus; HBV, hepatitis B virus; HCV, hepatitis C virus; HIV, human immunodeficiency virus; HNSCC, head and neck squamous cell carcinoma; NPC, nasopharyngeal carcinoma; GC, gastric cancer; HCC, hepatocellular carcinoma; NSCLC, non-small cell lung cancer; TME, tumor microenvironment; TIL, tumor-infiltrating lymphocyte; TLS, tertiary lymphoid structure; PD-1, programmed cell death protein 1; PD-L1, programmed death-ligand 1; CTLA-4, cytotoxic T-lymphocyte–associated antigen 4; ICI, immune checkpoint inhibitor; irAE, immune-related adverse event. OI, opportunistic infection.

This table summarizes the major tumor types, key viral oncogenic mechanisms, immune microenvironment characteristics, immune checkpoint features, reported ICIs response patterns, and safety considerations for five major oncogenic or clinically relevant viruses.

### Rethinking clinical management: shifting from feasibility to optimization

**Figure 3:**
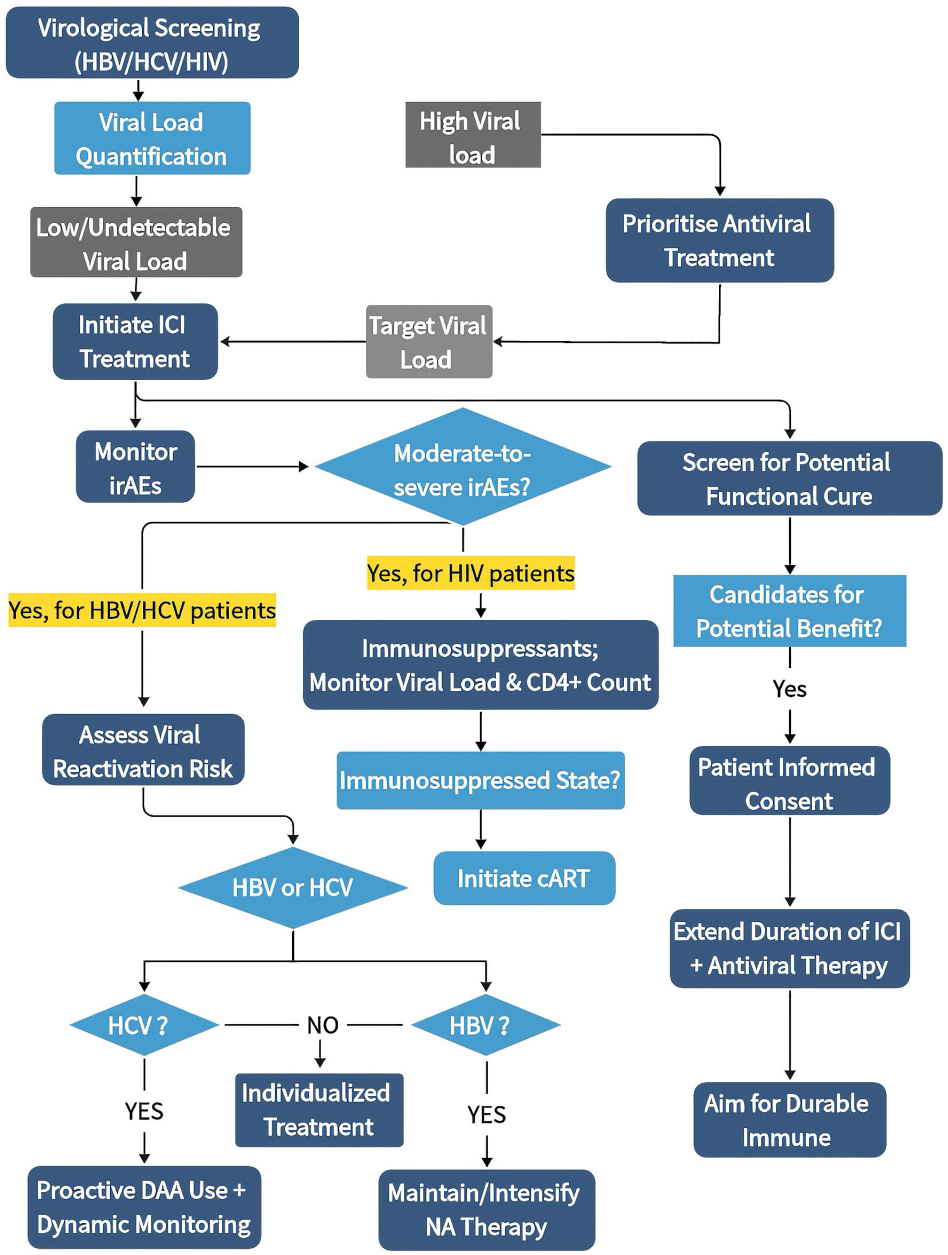
Guiding principles and strategic directions for combination ICIs therapy in patients with HBV, HCV, or HIV. In patients with HBV, HCV, or HIV, we propose several guiding principles for clinical management and future trial design: (1) Virological Optimization Prior to Treatment: Whenever feasible, effective virological control—defined as reducing HBV DNA, HCV RNA, or HIV RNA to low or undetectable levels—should be achieved before the initiation of ICIs. This approach aims to minimize persistent antigenic stimulation and mitigate the risk of viral reactivation. (2) Integrated Management of irAEs: Should moderate-to-severe irAEs arise that necessitate systemic immunosuppression, the risk of viral reactivation must be integrated into the clinical decision-making process. This entails maintaining or intensifying NA therapy for HBV patients and adopting a more proactive use of DAAs for HCV patients, coupled with longitudinal virological monitoring. (3) Exploration of Virological Control: In selected patients with favorable baseline virological features (e.g., those with low baseline HBV DNA or a sustained downward trend in HBsAg titers), an extended overlapping period of ICIs and antiviral therapy may be explored under rigorous monitoring and informed consent. Such strategies aim to investigate the feasibility of achieving more durable immunological remission.

In cancer patients with concurrent HBV, HCV, or HIV, clinical discussions regarding ICIs have long been centered predominantly on safety. The core concerns often revolve around whether such therapy will trigger viral reactivation or severe irAEs, which restricts clinical decision-making to the rudimentary question of “feasibility.” We contend that it is necessary—while remaining premised on safety—to explore how to deploy ICIs, antiviral therapy, and immunosuppressive strategies in more rational sequences and combinations across diverse viral contexts. This approach aims to achieve an optimal equilibrium between therapeutic efficacy and risk management. Whenever feasible, effective virological control—characterized by reducing HBV DNA, HCV RNA, or HIV RNA to low or even undetectable levels—should be achieved prior to the initiation of ICIs therapy to mitigate the risks of persistent antigenic stimulation and potential viral reactivation. Furthermore, should moderate-to-severe irAEs necessitating systemic immunosuppression arise during ICIs treatment, the risk of viral reactivation must be integrated into the global clinical decision-making process. Specifically, patients with HBV infection should continue or intensify nucleos(t)ide analog (NA) therapy, while those with HCV infection may benefit from a more proactive integration of DAAs, complemented by longitudinal virological monitoring. Additionally, for subgroups of patients exhibiting potential for a “functional cure”—such as individuals with low baseline viral loads or those demonstrating a sustained downward trend in HBsAg titers during treatment—exploring extended overlapping periods of ICIs and antiviral regimens may be warranted to assess the feasibility of achieving more durable immunological remission (**Figure 3**).

## Limitations and future directions

Another important consideration is the potential impact of heterogeneity and confounding factors when interpreting the efficacy of ICIs in virus-associated HCC. In particular, for HBV- or HCV-related HCC, several clinical and epidemiological variables may substantially influence patient outcomes. These include the stage of underlying cirrhosis, prior exposure to TKIs, regional differences in patient cohorts (e.g., Asian versus Western populations), and viral genotypes. Such factors may independently affect prognosis and treatment response, thereby potentially confounding comparisons between viral and non-viral HCC in terms of ICIs benefit. Therefore, caution is warranted when interpreting cross-study differences, and future studies with more standardized stratification and adjustment for these variables are needed to better clarify the true impact of viral etiology on immunotherapy outcomes.

Beyond these disease-specific considerations, several broader limitations of the current literature should also be acknowledged. This review primarily relies on retrospective cohorts, small-cohort studies, and real-world data; as such, the level of evidence remains limited, and certain conclusions are exploratory and hypothetical in nature. Furthermore, the substantial heterogeneity across diverse viral etiologies, tumor types, treatment lines, and combination regimens precludes the formulation of definitive operational guidelines at present. Moving forward, the following research priorities are warranted: first, prospective, stratified randomized controlled trials specifically tailored for virus-associated malignancies should be designed. These studies must clearly define viral status, TME characteristics, and molecular tumor subtypes at enrollment to facilitate the discovery of early predictive biomarkers for viral reactivation. Second, it is essential to integrate virological endpoints (e.g., viral load, antigen profiles, and functional cure rates) concurrently with oncological endpoints (e.g., ORR, PFS, OS, and irAEs) within the clinical study design.

In conclusion, virus-associated malignancies do not constitute a “marginalized population” in the era of ICIs. Accumulating evidence suggests that the interplay among infection, immunity, and cancer may help refine the clinical use of ICIs and improve our understanding of treatment response in these settings. In some contexts, such insights may also inform future strategies for the control of chronic viral infections, although their therapeutic implications remain to be validated. Overall, this evolving intersection between cancer immunology and infection immunology highlights an important direction for future translational and clinical research.

## Glossary

AASLD: American Association for the Study of Liver Diseases
AGEJ: Adenocarcinoma of the gastroesophageal junction
ALT: Alanine aminotransferase
AST: Aspartate aminotransferase
AEs: Adverse events
BL: Burkitt lymphoma
BOR: Best overall response
B2M: β2-microglobulin
cART: Combination antiretroviral therapy
CI: Confidence interval
CMV: Cytomegalovirus
CR: Complete response
CPS: Combined positive score
CTLA-4: Cytotoxic T-lymphocyte–associated protein 4
DAA: Direct-acting antiviral
DCR: Disease control rate
DLBCL: Diffuse large B-cell lymphoma
DOR: Duration of response
EBNA: Epstein–Barr nuclear antigen
EBV: Epstein–Barr virus
FFS: Failure-free survival
GC: Gastric cancer
HBsAg: Hepatitis B surface antigen
HBV: Hepatitis B virus
HBVr: Hepatitis B virus reactivation
HCC: Hepatocellular carcinoma
HCV: Hepatitis C virus
HCVr: Hepatitis C virus reactivation
HFSR: Hand-foot skin reaction
HHV-4: Human herpesvirus 4
HIV: Human immunodeficiency virus
HNSCC: Head and neck squamous cell carcinoma
HR: Hazard ratio
HPV: Human papillomavirus
ICIs: Immune checkpoint inhibitors
IFN-γ: Interferon gamma
irAEs: Immune-related adverse events
IRIS: Immune reconstitution inflammatory syndrome
LAG-3: Lymphocyte activation gene 3
LAGC: Locally advanced gastric cancer
MDSCs: Myeloid-derived suppressor cells
NA: nucleos(t)ide analog therapy
MHC-I: Major histocompatibility complex class I
NKTL: Natural killer/T cell lymphoma
NPC: Nasopharyngeal carcinoma
NSCLC: Non–small cell lung cancer
OI: Opportunistic infection
OPSCC: Oropharyngeal squamous cell carcinoma
OR: Odds ratio
ORR: Objective response rate
OS: Overall survival
PD-1: Programmed cell death protein 1
PD-L1: Programmed death-ligand 1
PFS: Progression-free survival
PR: Partial response
PTLD: Post-transplant lymphoproliferative disorder
SAT: Standardized antiviral therapy
SD: Stable disease
STING: Stimulator of interferon genes
SVR: Sustained virologic response
TILs: Tumor-infiltrating lymphocytes
TIL-Bs: Tumor-infiltrating B cells
TME: Tumor microenvironment
TLS: Tertiary lymphoid structures
TRAEs: Treatment-related adverse events
Tregs: Regulatory T cells
Trm: Tissue-resident memory T cells
VEGF: Vascular endothelial growth factor
VEGFR: Vascular endothelial growth factor receptor
WBC: White blood cell
WHO: World Health Organization.
