# Supplementary material for: Virus-driven remodeling of the immune microenvironment and response to immune checkpoint inhibitors: the infection– immunity–cancer crosstalk
Source: Front Immunol. 2026 Apr 27;17:1807408. doi: 10.3389/fimmu.2026.1807408 (PMC13158212; doi:10.3389/fimmu.2026.1807408)
Supplement: Supplementary file 1 [file Table1.docx]

| **Virus** | **Tumor type** | **Ref.** | **Study design** | **Evidence level** | **Sample size** | **Treatment line** | **Disease status** | **Regimen** | **Comparator** | **Primary endpoint / reported outcome type** |
| --- | --- | --- | --- | --- | --- | --- | --- | --- | --- | --- |
| HPV | HNSCC | [15] | Phase 1b trial | Prospective evidence | n= 60 | Previously treated / mixed lines | Recurrent or metastatic | Pembrolizumab monotherapy | None (single-arm) | ORR, PFS, OS, safety |
| HPV | HNSCC | [18] | Randomized Phase III (CheckMate 141) | Randomized evidence | n=361 | Second-line | 3/4 | Nivolumab | Investigator's choice | OS |
| HPV | HNSCC | [19] | phase II study | Prospective evidence | n=171 | Previously treated; platinum- and cetuximab-refractory | Recurrent/metastatic | Pembrolizumab 200 mg every 3 weeks | None (single-arm) | ORR, duration of response, PFS, OS, safety; subgroup outcomes by HPV and PD-L1 status |
| HPV | HNSCC | [20] | Randomized Phase II | Randomized evidence | n=30 | Neoadjuvant | 1/2 | Nivolumab | None | Immunological response |
| HPV | HNSCC | [33] | Retrospective Cohort | Retrospective evidence | n=84 | Multiple lines | 3/4 | Anti-PD-1/L1 | None | TLS maturity / PFS |
| HPV | HNSCC | [39] | Systematic review | Mixed evidence | 11 studies; n=791 | ICI-treated population | Recurrent/metastatic | PD-1 inhibitors (mainly nivolumab and pembrolizumab | Not a uniform comparator study | Treatment-related adverse events, potential irAEs |
| HPV | LACC | [42] | Phase Ib trial | Prospective evidence | n=30 | First-line | FIGO 2018 stage IB3–IVA, locally advanced disease | Toripalimab + concurrent chemoradiotherapy (pelvic EBRT + brachytherapy + weekly cisplatin) | None (single-arm) | Safety, ORR, PFS, late radiation-related toxicities |
| EBV | Relapsed/refractor NKTL | [70] | Retrospective multicenter case series | Case-based evidence | n=7 | Relapsed/refractory after L-asparaginase–containing regimens | recurrent/refractory | Pembrolizumab 2 mg/kg every 3 weeks in most patients | None (single-arm) | Objective response, CR/PR, duration of remission/follow-up, radiologic response, molecular response (circulating EBV DNA), safety |
| EBV | DLBCL | [72] | Multicentre, single-arm, phase II trial | Prospective evidence | n=28 | First-line | Stage II–IV | Sequential avelumab + rituximab priming, followed by R-CHOP, then avelumab consolidation (AvR-CHOP) | None (single-arm) | Grade ≥3 irAEs, ORR, CR rate, failure-free survival, overall survival, PET/radiomic and biomarker correlates |
| EBV | NPC | [75] | Multicenter, open-label, single-arm, phase II trial | Prospective evidence | n=23 | Previously treated; post-platinum and post-second-line chemotherapy | Recurrent/metastatic | Cadonilimab monotherapy 6 mg/kg Q2W | None (single-arm) | ORR, DCR, DoR, TTR, PFS, OS, safety; subgroup outcomes by PD-L1 and plasma EBV-DNA level |
| EBV | NPC | [76] | phase II trial | Prospective evidence | n=40 | Previously treated; post-first-line chemotherapy | Recurrent/metastatic EBV-positive NPC | Nivolumab 3 mg/kg every 2 weeks + ipilimumab 1 mg/kg every 6 weeks | None (single-arm) | BOR/ORR, clinical benefit rate, PFS, OS, duration of response, time to progression, safety; exploratory biomarker analyses including plasma EBV-DNA |
| EBV | GC | [78] | Single-center, prospective phase II trial with integrated molecular profiling | \| Prospective  evidence \| \| --- \|  \|  \| \| --- \| | n=61 | Second-line or third-line salvage treatment | Metastatic | Pembrolizumab monotherapy | None (single-arm) | ORR, DCR, duration of response, PFS, molecular correlates of response, ctDNA dynamics |
| EBV | CG | [79] | Single-center retrospective cohort study | Retrospective evidence | n=305 | First-line | Initially metastatic, recurrent, or locally advanced unresectable | Nivolumab plus CAPOX or FOLFOX chemotherapy | Chemotherapy alone (fluoropyrimidine + platinum doublet) in an EBV-positive control cohort | PFS, OS, ORR, multivariable HRs, subgroup outcomes by PD-L1 CPS |
| HBV + EBV | NSCLC | [85] | Case report | Case-based evidence | n=1 | ICI-treated setting; after chemo-immunotherapy | Advanced | Albumin-bound paclitaxel + carboplatin + tislelizumab; colitis treated with oral methylprednisolone, mesalazine, and supportive care | None | Clinical improvement/resolution of diarrhea; endoscopic findings; pathological findings; follow-up laboratory and endoscopic/pathologic assessments |
| CMV + EBV | Solid tumors (eg. breast cancer) | [86] | Case report | Case-based evidence | n=2 | ICI-treated setting; steroid-refractory immune-related colitis after pembrolizumab-containing therapy | Solid tumors with severe/refractory ICI-related colitis | Pembrolizumab-containing cancer therapy; colitis managed with steroids ± infliximab, plus oral valganciclovir after viral detection | None | Clinical response of colitis, endoscopic findings, histopathology, tissue CMV/EBV PCR, symptom resolution after antiviral therapy |
| EBV | UGICs | [87] | Systematic review and meta-analysis | prospective evidence | 36 studies; n=12,440 | Mixed: first-line and second-line or later | Advanced / metastatic | ICI monotherapy and ICI-based combination therapy | Chemotherapy, or none in single-arm studies, depending on included trial | ORR, PFS, OS; pooled ORs and HRs; subgroup analyses by PD-L1, EBV, MSI, region, sex, treatment line, and regimen |
| HBV | HCC | [90] | Systematic review and meta-analysis of phase II/III trials | Mixed evidence | n= 5646 | first line | Advanced/unresectable | ICI Combination/monotherapy | TKIs (sorafenib or lenvatinib) | PFS/OS/ORR by etiology subgroup |
| HBV | HCC | [91] | Systematic review and meta-analysis of randomized controlled trials | Meta-analyzed randomized evidence | n=5316 | First-line | Mostly locally advanced, metastatic, and/or unresectable HCC | PD-1/PD-L1–based immunotherapy alone or combined with TKIs/anti-VEGF agents | Mainly sorafenib or other non-ICI control arms, depending on trial | ORR, PFS, OS; pooled subgroup outcomes by viral etiology |
| HBV | HCC | [92] | Phase 2 clinical trial | Prospective evidence | n=30 | First-line | 2/3 | Durvalumab + Tremelimumab | None (single-arm) | Safety (HBVr) and Efficacy (ORR) |
| HBV | HCC | [95] | Phase 1 dose escalation trial | Prospective evidence | n=15 | Adjuvant (Post-op) | HBV-related HCC after curative-intent surgical resection | Sequential Ropeg-IFNα-2b then anti-PD-1 (nivolumab) | None (single-arm) | Safety, dose-limiting toxicity, HBsAg loss/reduction, anti-HBs induction, recurrence-free survival, overall survival |
| HBV | Solid tumors excluding HCC | [99] | Real-life retrospective single-center cohort study | Retrospective real-world evidence | n=150 | Mixed ICI-treated setting | Mostly stage 4 | ICI monotherapy or chemo-immunotherapy; mainly pembrolizumab, nivolumab, and atezolizumab | No uniform comparator arm | Prevalence of potential occult HBV infection (pOBI), HBV reactivation incidence, ALT/hepatic flare, virologic monitoring outcomes |
| HBV | Advanced cancers (predominantly HCC; NSCLC, and others) | [100] | Systematic review of case reports, case series, and prospective trials | Mixed evidence | 89 HBV patients | Mixed lines of therapy | Advanced-stage cancers | Mostly PD-1 inhibitor monotherapy; also CTLA-4 monotherapy, PD-1/CTLA-4 combinations, and rare PD-L1 inhibitor use | None; no uniform comparator arm across included studies | Hepatic transaminase elevation, viral load change/reactivation, virus-related hepatitis, ORR by tumor type, descriptive safety and efficacy outcomes |
| HBV | Solid tumors | [105] | Systematic Review and Meta-Analysis | Mixed evidence | n=  2561 | Multiple lines | 3/4 | Various ICIs | Prophylaxis vs. No prophylaxis | HBV reactivation (HBVr) rate |
| HBV | HCC | [108] | Retrospective, real-world study | Retrospective evidence | n=152 | First-line | 2/3 | Lenvatinib + Sintilimab | Lenvatinib monotherapy | OS/PFS/DCR |
| HBV | HCC | [113] | Systematic Review and Network Meta-Analysis | Randomized evidence | n=  2155  (subgroup) | First-line | 2/3 | ICI + anti-VEGF/VEGFR or Dual ICI | Sorafenib or Lenvatinib | OS/PFS hierarchy |
| HBV | Mixed cancers | [121] | Historical cohort study | Retrospective real-world evidence | n=3465positive n=511  negative n=2954 | Mixed ICI-treated setting | receiving ICIs | PD-1/PD-L1/CTLA-4 inhibitors, mostly monotherapy | internal comparisons by HBsAg status, HCC status, and antiviral prophylaxis | HBV reactivation, grade 3–4 hepatitis/hepatic adverse events, HBsAg seroclearance |
| HBV | Mixed cancers (HCC) | [122] | Retrospective single-center cohort study | Retrospective real-world evidence | n=162 | Mixed ICI-treated setting; mostly previously treated | HBsAg/ HBcAb positive cancer patients receiving ICIs | PD-1/PD-L1 inhibitor monotherapy or combination therapy | Internal comparisons by antiviral prophylaxis status and serologic subgroup | HBV reactivation incidence, hepatic adverse events, grade 3–4 hepatitis, treatment disruption, HBsAg clearance |
| HBV | HCC | [124] | Retrospective–prospective cohort study | Real-world prospective/retrospective evidence | n=60 | Mixed | Intermediate-stage TACE-refractory or advanced unresectable HCC | Nivolumab or pembrolizumab | Internal comparison by baseline HBV DNA and nucleos(t)ide analogue (NUC) status | HBV reactivation, HBV DNA kinetics, ALT flare/hepatitis, immune-related hepatitis, tumor response |
| HBV | Advanced cancers (predominantly HCC) | [125] | Systematic review with supplemental FAERS case identification | Mixed evidence | n=633 | Mixed ICI-treated setting | Advanced cancers | PD-1/PD-L1/CTLA-4–based ICI therapy | None | HBV reactivation, HBV-DNA kinetics, liver enzyme abnormalities, HBV-related outcomes, descriptive antitumor responses |
| HBV | HCC | [126] | Retrospective territory-wide cohort study | Retrospective real-world evidence | n=1596 | First systemic treatment comparison (ICI vs TKI), with additional analysis among ever-ICI patients by ICI type | Advanced liver cancer with current or past HBV infection | Pembrolizumab, atezolizumab–bevacizumab, nivolumab–ipilimumab; | TKI-treated cohort for primary comparison; secondary comparison across ICI regimens | HBV reactivation (AASLD and APASL criteria), hepatitis flare, hepatic decompensation, HBsAg seroclearance; adjusted hazard estimates for HBVr risk |
| HBV | HCC | [128] | Retrospective single-center cohort study | Retrospective real-world evidence | n=218 | Mixed ICI-treated setting | Mostly advanced disease | PD-1 inhibitor monotherapy or PD-1 inhibitor + angiogenesis inhibitor combination; all patients received first-line NA prophylaxis (ETV/TDF/TAF) | Internal comparison between anti–PD-1 monotherapy and anti–PD-1 plus angiogenesis inhibitor combination therapy | HBV reactivation incidence, hepatitis/hepatic flare, HBV-associated hepatitis, HBsAg seroclearance, risk factors for HBVr |
| HCV | HCC | [90] | Systematic review and meta-analysis of phase II/III trials | Mixed evidence | n=5,646 | First-line | Advanced/unresectable | ICI Combination/monotherapy | TKIs (sorafenib or lenvatinib) | OS, PFS, ORR by etiology subgroup |
| HCV | HCC | [91] | Systematic review and meta-analysis of randomized controlled trials | Meta-analyzed randomized evidence | n=5316 | First-line | Mostly locally advanced, metastatic, and/or unresectable HCC | PD-1/PD-L1–based immunotherapy alone or combined with TKIs/anti-VEGF agents | Mainly sorafenib or other non-ICI control arms, depending on trial | ORR, PFS, OS; pooled subgroup outcomes by viral etiology |
| HCV | Advanced cancers (predominantly HCC; NSCLC, and others) | [100] | Systematic review of case reports, case series, and prospective trials | Mixed evidence | 98 HCV-infected patients | Mixed lines of therapy | Advanced-stage cancers | Mostly PD-1 inhibitor monotherapy; also CTLA-4 monotherapy, PD-1/CTLA-4 combinations, and rare PD-L1 inhibitor use | None; no uniform comparator arm across included studies | Hepatic transaminase elevation, viral load change, virus-related hepatitis, ORR by tumor type, descriptive safety and efficacy outcomes |
| HCV | HCC | [113] | Systematic Review and Network Meta-Analysis | Randomized evidence | n=785 (Subgroup) | First-line | 2/3 | ICI + anti-VEGF/VEGFR or Dual ICI | Sorafenib or Lenvatinib | OS/PFS hierarchy |
| HCV | Solid tumors | [116] | Prospective observational study | Prospective evidence | n=52 | Mixed: first-line and salvage ICI therapy | Advanced/progressive solid tumors | Anti–PD-1/PD-L1/CTLA-4–based therapy | None | HCV inhibition, HCV reactivation, safety, grade 3–4 AEs, hepatitis flare, SVR12/SVR24 in DAA-treated patients |
| HCV | Advanced cancers (most commonly NSCLC and HCC) | [117] | Single-institution retrospective chart review | Retrospective real-world evidence | n=40 | Mixed ICI-treated setting | Cancer of any type and stage; many had advanced/metastatic disease | Nivolumab, pembrolizumab, atezolizumab, durvalumab, or nivolumab + ipilimumab | None | irAEs, hepatotoxicity, pneumonitis, colitis, fatigue, hypothyroidism |
| HIV | Advanced-stage cancers (mixed tumor types) | [130] | Systematic review | Mixed evidence | n=73 | Mostly previously treated ICI-treated population; prior systemic therapy common | Advanced-stage | Anti–PD-1, anti–CTLA-4, anti–PD-1/CTLA-4 combination, or sequential ipilimumab/nivolumab | None | Grade ≥3 irAEs, HIV viral load change, CD4 cell count change, ORR by tumor type, descriptive safety and efficacy outcomes |
| HIV | Advanced cancers (mixed tumor types) | [131] | Retrospective multicenter real-world cohort study | Real-world retrospective evidence | n=390 | Mixed; first-line and later-line ICI therapy | Advanced/metastatic | Anti–PD-1/anti–PD-L1 monotherapy, anti–PD-1/anti–PD-L1 + chemotherapy, anti–PD-1/anti–PD-L1 + targeted therapy, or anti–PD-1 + anti–CTLA-4 | No uniform comparator in overall cohort | irAEs, OS, PFS, ORR, HIV viral load change, CD4+ T-cell count change; matched RMST comparisons for OS and PFS in mNSCLC |
| HIV | Advanced cancers (mixed tumor types) | [132] | Prospective observational real-world cohort study | Prospective real-world evidence | n=140 | Mixed ICI-treated setting; treatment line not uniform | Cancer population with HIV infection, mostly advanced disease receiving ICIs | Mainly anti–PD-1; also anti–PD-L1, anti–PD-1 + anti–CTLA-4, and anti–PD-1 + anti-VEGFR regimens | None | Grade ≥3 irAEs, incidence rates, cumulative incidence, risk factors for severe irAEs, survival outcomes by cancer type |
| HIV | NSCLC | [133] | Single-center comparative clinical study | Prospective comparative evidence | n=58 (positive =18; negative =40) | First-line | Unresectable locally advanced or advanced NSCLC, AJCC 8th stage IIIC–IV | Tislelizumab 200 mg + platinum-based chemotherapy for 4–6 cycles, followed by tislelizumab maintenance | HIV-negative control group receiving the same tislelizumab + platinum-based chemotherapy regimen | ORR, DoR, PFS, OS, safety, CD4+ T-cell count changes |
